# Effects of a Macroporous Resin Extract of *Dendrobium officinale* Leaves in Rats with Hyperuricemia Induced by Anthropomorphic Unhealthy Lifestyle

**DOI:** 10.1155/2023/9990843

**Published:** 2023-01-04

**Authors:** Lin-Zi Li, Xi-Ming Wang, Xiao-Jie Feng, Kun Liu, Bo Li, Li-Jie Zhu, Wan-Feng Xu, Xiang Zheng, Ying-Jie Dong, Xing-Lishang He, Hao-Ru Guan, Yan-Yan Ding, Han-Song Wu, Chuan-Jie Zhou, Sen-Yu Ye, Bei-Bei Zhang, Gui-Yuan Lv, Su-Hong Chen

**Affiliations:** ^1^Zhejiang Provincial Key Laboratory of TCM for Innovative R & D and Digital Intelligent Manufacturing of TCM Great Health Products, Zhejiang University of Technology, Hangzhou, Zhejiang 310014, China; ^2^Zhejiang Senyu Co., Ltd., Yiwu, Zhejiang 322099, China; ^3^Center for Food Evaluation, State Administration for Market Regulation, No. 188 Western Road of South Fourth Ring Road, Fengtai District, Beijing 100070, China; ^4^College of Pharmaceutical Science, Zhejiang Chinese Medical University, Hangzhou, Zhejiang 310053, China

## Abstract

**Aim:**

Hyperuricemia (HUA) has received increased attention in the last few decades due to its global prevalence. Our previous study found that administration of a macroporous resin extract of *Dendrobium officinale* leaves (DoMRE) to rats with HUA that was induced by exposure to potassium oxazine combined with fructose and a high-purine diet led to a significant reduction in serum uric acid (SUA) levels. The aim of this study was to explore the effects of DoMRE on hyperuricemia induced by anthropomorphic unhealthy lifestyle and to elucidate its possible mechanisms of action.

**Methods:**

Dosages (5.0 and 10.0 g/kg/day) of DoMRE were administered to rats daily after induction of HUA by anthropomorphic unhealthy lifestyle for 12 weeks. The levels of UA in the serum, urine, and feces; the levels of creatinine (Cr) in the serum and urine; and the levels of aspartate aminotransferase (AST) and alanine aminotransferase (ALT) in serum were all measured using an automatic biochemical analyzer. The activities of xanthine oxidase (XOD) and adenosine deaminase (ADA) in the serum, liver, and intestine tissue supernatant were measured using appropriate kits for each biological target. The expressions levels of UA transporters (ABCG2 and GLUT9), tight junction (TJ) proteins (ZO-1 and occludin), and inflammatory factors (IL-6, IL-8, and TNF-*α*) in the intestine were assayed by immunohistochemical (IHC) staining. Hematoxylin and eosin (H&E) staining was used to assess histological changes in the renal and intestinal tissues.

**Results:**

DoMRE treatment significantly reduced SUA levels and concomitantly increased fecal UA (FUA) levels and the fractional excretion of UA (FEUA) in HUA rats. Furthermore, DoMRE significantly reduced both the XOD activity in the serum, liver, and intestine and the ADA activity in the liver and intestine. DoMRE also effectively regulated the expression of GLUT9 and ABCG2 in the intestine, and it significantly upregulated the expression of the intestinal TJ proteins ZO-1 and occludin. Therefore, DoMRE reduced the damage to the intestinal barrier function caused by the increased production of inflammatory factors due to HUA to ensure normal intestinal UA excretion.

**Conclusion:**

DoMRE demonstrated anti-HUA effects in the HUA rat model induced by an anthropomorphic unhealthy lifestyle, and the molecular mechanism appeared to involve the regulation of urate transport-related transporters (ABCG2 and GLUT9) in the intestine, protection of the intestinal barrier function to promote UA excretion, and inhibition of XOD and ADA activity in the liver and intestine to inhibit UA production in the HUA-induced rats.

## 1. Introduction

Hyperuricemia (HUA) is a common metabolic disease characterized by elevated blood uric acid levels and is associated with aberrant purine metabolism in humans [1]. HUA is a common risk factor for diabetes, hypertension, obesity, and other diseases, and the increased levels of uric acid (UA) in the blood can promote the deposition of urate crystals in tissues, arousing several pathological conditions, such as acute gouty arthritis, urolithiasis, and obstructive uropathy [2], which can seriously endanger human health. Epidemiological survey results showed that the incidence of hyperuricemia in China has increased significantly in recent years to 11.1% [3].

Urate levels in the blood are regulated by the balance between the formation and excretion of UA [4]. Therefore, disruption of this equilibrium due to decreased excretion, increased synthesis, or both will lead to elevated plasma UA concentrations, resulting in HUA. Two-third of all urate produced in the body is cleared renally and excreted from the body through the urine, while the remaining one-third undergoes extra-renal excretion via the gut. Clinically, approximately 90% of patients with primary HUA suffer from poor UA excretion [5]. Furthermore, approximately 90% of HUA patients with gout suffer from poor renal excretion of UA; some patients have been found to excrete only 40% of urate renally. In these situations, intestinal elimination might compensate for the lack of renal clearance capacity [6]. Therefore, the gastrointestinal tract plays an important role in maintaining urate homeostasis and might be a potential therapeutic target for HUA.

During the past few decades, changes in diet structure, such as consuming foods high in purines, and lifestyle have promoted increases in the prevalence and incidence of HUA throughout the world. For example, the incidence of HUA in China has increased about tenfold in the past 40 years [7]. Studies have shown that HUA is caused by lifestyle changes, drug use, diseases, genetics, and other factors. Among these factors, unhealthy lifestyle habits, such as irregular work and sleep schedules, consuming high-fat and high-sugar diets, alcoholism, mental stress, and so on, represent one of the most significant contributors to the development of HUA. UA levels in the blood are closely related to emotion-related psychopathology (e.g., anxiety and depression). Psychological stress can affect neuroendocrine regulation, activate the hypothalamic-pituitary-adrenal axis (HPA axis), and affect purine metabolism and UA excretion [8, 9]. In addition, alcohol can increase the concentration of lactic acid in the body, which can lead to the inhibition of UA excretion in the renal tubules [10].

At present, genotypic and phenotypic changes in various models of HUA are studied by supplementing exogenous UA or UA precursors, inhibiting uricase activity, and inhibiting renal UA excretion; however, these are not entirely characteristic of the pathogenesis of human primary HUA. Therefore, it is of great significance to establish an animal model of HUA that is more relevant to human UA metabolism and pathogenesis to better study the pathogenesis of HUA and evaluate the therapeutic efficacy of drugs to treat HUA. Because living an unhealthy lifestyle can lead to significant increases in UA production in humans, this study was aimed at establishing a HUA rat model, in which the HUA was induced based on anthropomorphic factors that can contribute to an unhealthy lifestyle, such as irregular work and sleep schedules, high mental stress, overeating, and excessive drinking. Therefore, the model entailed exposing the rat to chronic stress + drinking alcohol + feeding high sugar and high fat + potassium oxazine (oral gavage).

*Dendrobium officinale* Kimura et Migo is a rare orchid species that has a long history of being used as a traditional Chinese herb for its wide range of health benefits and because its leaves are edible. In 2020, Zhejiang Province promulgated the first local food safety standard about it. In our previous research, we confirmed that the macroporous resin extract of *Dendrobium officinale* leaves (DoMRE) reduced UA levels in rats with HUA induced by potassium oxazine combined with fructose and a high-purine diet [11, 12]. This study aimed to research the pharmacodynamics of the DoMRE and elucidate its anti-hyperuricemia mechanism through the XOD/ADA system and UA excretion (renal excretion and intestine excretion) pathway by establishing a rat model of HUA in which the rats were exposed to indicators of an anthropomorphic unhealthy lifestyle. This study may lay the foundation for the development of new drugs for treating hyperuricemia.

## 2. Materials and Methods

### 2.1. Materials and Reagents

*Dendrobium officinale* leaves were provided by Senyu Holding Group (Zhejiang, China), the origin is Zhejiang (Yiwu, Zhejiang) and three years old. ADS-17 macroporous adsorption resin was purchased from Ganzhou Baoen Adsorption Material Technology Co., Ltd. (Hebei, China), fructose powder was purchased from Xiwang Pharmaceutical Co., Ltd. (Shandong, China), cholesterol was purchased from Xiangsheng Biotechnology Co., Ltd. (Jiangsu, China), sodium cholate was purchased from Yanao Biological Technology Co., Ltd. (Hebei, China), lard oil powder was purchased from Huatai Oil & Fat Co., Ltd. (Qingdao, China), and alcohol was purchased from Beijing Red Star Co., Ltd (Beijing, China). Uric acid (UA) (200529201), aspartate aminotransferase (AST) (200818201), alanine aminotransferase (ALT) (200910201), and creatinine (Cr) (200826101) biochemical reagents kits were purchased from Ningbo Meikang Biotechnology Co., Ltd. (Zhejiang, China), and potassium oxazine (Y18M11C113291) and hematoxylin-eosin (HE) dye solution (J22D9Y78310) were obtained from Shanghai Yuanye Biotechnology Co., Ltd (Shanghai, China).

XOD assay kit (20210120) and ADA assay kit (20210125) were purchased from Nanjing Jiancheng Technology Co., Ltd (Jiangsu, China). The rabbit polyclonal antibodies, including ATP-binding cassette superfamily G member 2 (ABCG2), zonula occludens 1 (ZO-1), occludin, interleukin-1*β* (IL-1*β*), interleukin-6 (IL-6), tumor necrosis factor-*α* (TNF-*α*), and glucose transporter 9 (GLUT9) mouse monoclonal antibody were purchased from Protein Technology Inc (MA, USA).

### 2.2. Preparation of Macroporous Resin Extract of *Dendrobium officinale* Leaves (DoMRE)

The preparation of the DoMRE was performed as previously described [12]. Firstly, dried *Dendrobium officinale* leaves were extracted three times with 75% (v/v) ethanol in water. The combined extracts were concentrated under reduced pressure to afford the ethanol extract. The ethanol extract was then redissolved in water, and the resulting solution was extracted with petroleum ether and *n*-butanol. After removal of the *n*-butanol following extraction, the extraction filtrate of *n*-butanol was concentrated by rotary evaporation apparatus to make *n*-butanol volatilize completely and discard the black insoluble impurities, and the crude extract of *Dendrobium officinale* leaves was obtained.

Macroporous resin AB-8 with weak polarity was used to purify the crude extract of *Dendrobium officinale* leaves. Firstly, the macroporous resin AB-8 was soaked in 95% ethanol for 24 h to fully expand the resin. The resin was repeatedly cleaned with 95% ethanol until the filtrate was no longer turbid and then washed with distilled water until it had no alcohol flavor. Then, it was soaked in 4% NaOH for 24 h and washed with distilled water to neutral. Finally, it was soaked in 4% HCl for 24 h, washed with distilled water to neutral, and soaked in water for later use. The pretreated macroporous adsorption resin was eluted with deionized water until the effluent was nearly colorless and then with 10% (v/v) ethanol in water. The 50% ethanol aqueous solution (v/v) eluate was collected and concentrated under vacuum to afford the DoMRE for the following animal experiments [12]. The content of total flavonoids in DoMRE was 16.52 ± 0.02% as measured by sodium nitrite-aluminum nitrate colorimetry.

### 2.3. Preparation of the HUA Model and Drug Administration

32 specific pathogen-free (SPF) male Sprague-Dawley (SD) rats with a body weight of 200 ± 20 g were purchased from Zhejiang Academy of Medical Sciences (Hangzhou China) (license number: SCXK 2019-0002), and all rats were raised under standard environmental conditions, with free access to water and diet. All animal procedures were performed in accordance with Regulations of Experimental Animal Administration and approved by the ethics committee of Zhejiang University of Technology.

After 5 days of adaptive feeding, the experimental rats were randomly divided into 4 groups (*n* = 8) according to body weight: (1) normal control group (NC); (2) hyperuricemia model group (MC); (3) low dose of DoMRE treatment group (DoMRE-L, 3.5 mg/kg, equal to 5.0 g raw herbs/kg body weight); (4) high dose of DoMRE treatment group (DoMRE-L, 7.0 mg/kg, equal to 10.0 g raw herbs/kg body weight).

The hyperuricemia rat model group (MC) was subjected to chronic unpredictable stress, drinking alcohol, high-sugar and high-fat diets, and potassium oxazine administration by oral gavage (200 mg/kg) to establish the hyperuricemia model. There were six methods of chronic stress: exposure to a wet bed for 24 h, swimming in cold water (0–4°C) for 5 min, sleep deprivation for 24 h, tail-clamping for 30 min, deliberate fasting for 24 h, and being exposed to an overcrowded environment for 24 h. The stressors were induced randomly, with each stressor varying in frequency and time to prevent the animals from adapting to the stressor(s) (Table 1). The rats were fed with a high-sugar and high-fat diet (normal diet (77.25%), fructose (10%), edible lard (10%), cholesterol (2.50%), and bile salt (0.25%)), and Red Star Erguotou (the alcohol volume fraction gradually increased from 5% to 22%) was added to the drinking water of the rats; 4% alcohol was provided for four consecutive days at the beginning of experiments, after which the percentage was increased to 8% on the fifth day and then increased by 4% every three days until reaching 22% (Table 2). The rats were given the drug for 12 weeks while making the model, the NC and MC rats were given distilled water (1 mL/100 g weight), and the other groups rats were given the same volume/weight of medicine. At the end of the experiment, the rats fasted overnight and were anesthetized with pentobarbital the next day. Blood was collected from the abdominal aorta and processed to collect the serum, and kidney, liver, and intestinal tissue samples were collected, washed with cold saline, and stored at −80°C until analysis.

### 2.4. Determination of Biochemical Analysis in Blood

At the 8^th^ and 12^th^ week of administration, blood samples were taken from the ophthalmic venous plexus and allowed to clot for 30 min at 37°C water bath and then centrifuged at 3500 rpm for 10 min to obtain serum. One hour before the blood sample collection, all rats were given potassium oxazine solution by gavage. Then, the serum was separated to detect the biochemical indexes of UA, Cr, AST, and ALT by automatic biochemical analyzer (Hitachi 7020, Japan).

### 2.5. Measurement of the Biochemical Assays in Urine and Fecal Samples

Two days before the end of the experiment, rats from each group were placed in metabolic cage for 24 h to collect the urine and determine the volume of this urine. The urine creatinine (UCr) and urine uric acid (UUA) levels were detected by automatic biochemical analyzer. FEUA was calculated as follows:

(1) $$\begin{array}{c} FEUA\%=\frac{UUA\times SCr}{\left(SUA\times UCr\right)}\times 100\%\text{.} \end{array}$$

Fecal samples were collected during the 12^th^ week of the experiments and dried at 60°C in drying oven. Approximately 0.1 g of the dried feces was mixed with 4 times the volume of PBS solution, ultrasonicated for 10 min, and centrifuged at 3500 r/min for 15 min. The supernatant was collected, and then the fecal uric acid (FUA) levels were measured using an automatic biochemical analyzer (Hitachi 7020, Japan).

### 2.6. Hematoxylin and Eosin (H&E) Staining Observation of Kidney and Intestine

The kidney, duodenum, ileum, and colon were put into 4% formalin for fixation and then embedded in paraffin. After that, all specimens were sectioned to obtain 4 *μ*m thickness, stained with hematoxylin and eosin (H&E). Finally, a biological microscope was used to capture the images of these tissues.

### 2.7. Determination of XOD and ADA Levels in Serum, Liver, and Intestine

Serum and tissues of liver and intestine were taken out from the −80°C refrigerator in liquid nitrogen, and the tissues were homogenized and centrifuged at 3500 r/min for 10 min at 4°C to collect supernatant. Then, according to the manufacturer's protocol, the XOD and ADA levels of serum, liver, and intestine tissue supernatant were measured with detection kits.

### 2.8. Immunohistochemistry (IHC) Staining Observation

To determine the expression of urate transporters in the intestine, IHC was used as previously described. The specimens were incubated sequentially with antibody against ABCG2, GLUT9, ZO-1, occludin, IL-1*β*, IL-6, and TNF-*α*. Finally, the secondary antibody HRP conjugated goat anti-rabbit IgG was added. The tissues were observed by the biological microscope, and the protein expression levels were evaluated by semiquantitative analysis as integrated option density (IOD) in positive area of the microphotograph with the Image-Pro Plus analysis software.

### 2.9. Statistical Analysis

All experiment data were expressed as mean ± standard deviation (SD). SPSS 17 statistical software was used for data analysis. Statistical differences between groups were determined by Student's *t*-test or one-way analysis of variance (ANOVA). *P* < 0.05 was considered as a statistically significant difference, and GraphPad Prism 7.0 was used for graph analysis.

## 3. Results

### 3.1. DoMRE Reduces SUA Levels and Prevents Liver Injury in HUA Rats

**Figure 1:**
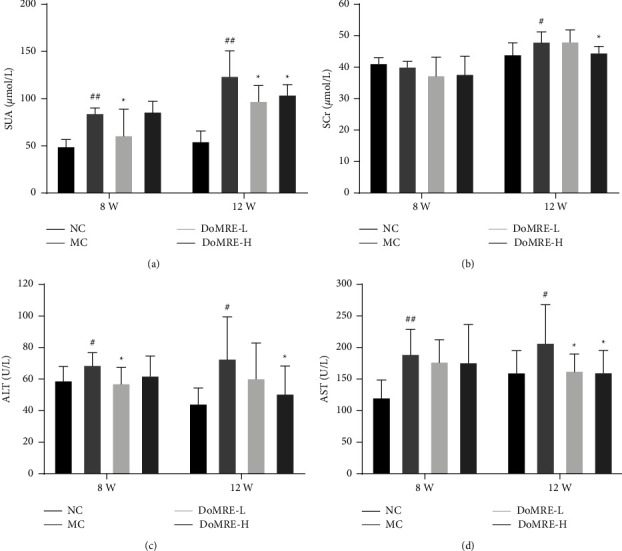
Effect of DoMRE on SUA and SCr levels. (a) SUA levels after 8 and 12-week oral administration. (b) SCr levels after 8 and 12-week administration. (c) ALT levels after 8 and 12-week administration. (d) AST levels after 8 and 12-week administration. NC, normal control group; MC, model control group; DoMRE-L, low dose of DoMRE group; DoMRE-H, high dose of DoMRE group. The data were expressed as mean ± SD of 8 rats in each group. ^#^*P* < 0.05; ^##^*P* < 0.01, compared with NC group; ^^*∗*^^*P* < 0.05; ^^*∗∗*^^*P* < 0.01, compared with MC group.

Serum uric acid (SUA) levels are considered an important indicator of HUA because UA is mainly produced by the liver and cleared by the kidneys and the intestine [13]. As shown in Figure 1(a), anthropomorphic unhealthy lifestyle for 8 weeks led to a significant augmentation in SUA levels relative to the normal control (NC) group (*P* < 0.01), which indicated that HUA model rats were established successfully. Throughout the 12-week modeling period, treatment with all doses of DoMRE significantly reduced the SUA levels of rats with anthropomorphic unhealthy lifestyle-induced HUA after 12 weeks (*P* < 0.05) (Figure 1(a)). These results suggested that DoMRE has the potential to significantly reduce SUA.

UA metabolism is closely related to the function of the liver, which is the main organ for performing de novo purine synthesis. Therefore, aspartate transaminase (AST) and alanine aminotransferase (ALT) are reliable indicators for the assessment of liver function [14]. The results are shown in Figures 1(c) and 1(d); after 12 weeks of administration, compared with the MC group, DoMRE-H could significantly reduce the ALT and AST levels (*P*  < 0.05), and DoMRE-L could significantly reduce the level of the AST (*P*  < 0.05).

### 3.2. DoMRE Prevents Renal Damage and Promotes Renal UA Excretion in the HUA Rats

Renal injury can be accompanied by an increase in serum creatinine (SCr) levels, which is an important indicator of renal dysfunction [15]. As shown in Figure 1(b), compared with the NC group, the SCr levels in MC group increased significantly after 12 weeks (*P* < 0.05). These results indicated that the modeling method of anthropomorphic unhealthy lifestyle could cause renal function injury in HUA model rats. At the same time, throughout the 12-week modeling period, DoMRE-H significantly decreased the SCr levels of rats (*P* < 0.05).

**Figure 2:**
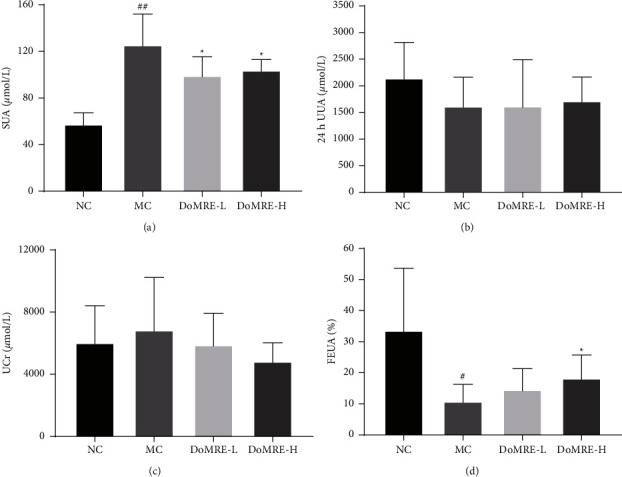
Effect of DoMRE on UV, 24 h UUA, UCr and FEUA levels and renal urate transporters protein levels. (a) SUA levels. (b) 24 h UUA levels. (c) UCr levels. (d) FEUA levels. NC, normal control group; MC, model control group; DoMRE-L, low dose of DoMRE group; DoMRE-H, high dose of DoMRE group. The data were expressed as mean ± SD of 8 rats in each group. ^#^*P* < 0.05; ^##^*P* < 0.01, compared with NC group; ^^*∗*^^*P* < 0.05; ^^*∗∗*^^*P* < 0.01, compared with MC group.

Renal dysfunction can affect UA excretion, and hyperuricemia aggravates kidney dysfunction [16]. The results are shown in Figure 2; compared with the NC group, the FEUA levels in MC group were significantly decreased after modeling for 12 weeks (*P* < 0.05, 0.01), and it was speculated that the modeling method of anthropomorphic unhealthy lifestyle could inhibit the ability of renal excretion of UA in HUA model rats. Compared with the MC group, there were significantly increased levels of FEUA in the DoMRE-H group. These results suggested that DoMRE promoted renal UA excretion and ameliorated renal dysfunction in the anthropomorphic unhealthy lifestyle-induced rat model of HUA to a certain extent.

### 3.3. DoMRE Decreases UA Formation by Regulating XOD and ADA Activity in HUA Rats

**Figure 3:**
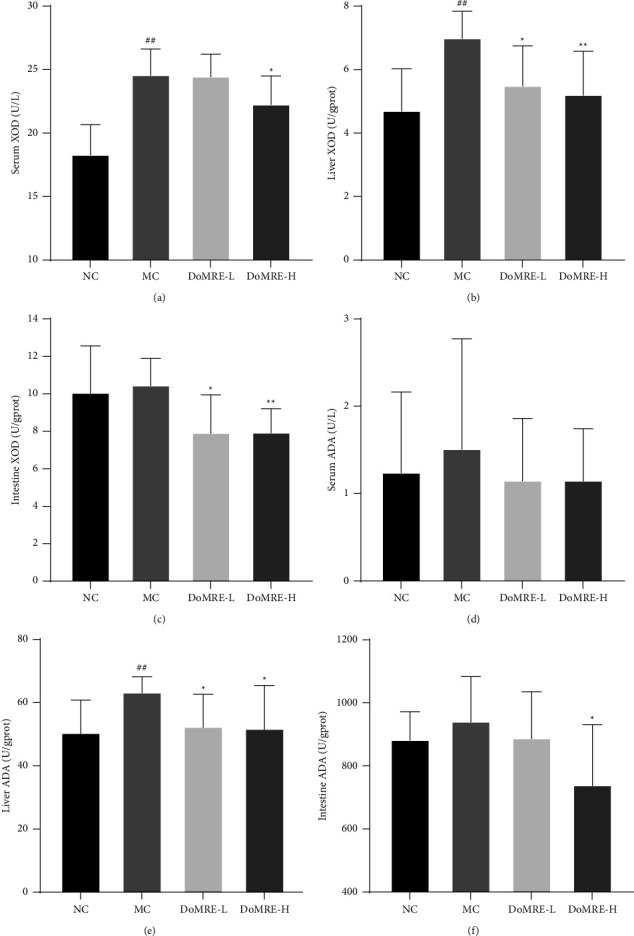
Effect of DoMRE on XOD and ADA activity. (a) The XOD activity in the serum after 12-week oral administration. (b) The XOD activity in the liver after 12-week oral administration. (c) The XOD activity in the small intestine after 12-week oral administration. (d) The ADA activity in the serum after 12-week oral administration. (e) The ADA activity in the liver after 12-week oral administration. (f) The ADA activity in the small intestine after 12-week oral administration. NC, normal control group; MC, model control group; DoMRE-L, low dose of DoMRE group; DoMRE-H, high dose of DoMRE group. The data were expressed as mean ± SD of 8 rats in each group. ^#^*P* < 0.05; ^##^*P* < 0.01, compared with NC group; ^^*∗*^^*P* < 0.05; ^^*∗∗*^^*P* < 0.01, compared with MC group.

Xanthine oxidase (XOD) and adenosine deaminase (ADA) are important enzymes in UA production and are found in the serum, liver, and small intestine [17]. The results are shown in Figures 3(a)–3(c); compared with the NC group, the XOD content in the liver and serum of the MC group increased significantly (*P* < 0.01). Compared with the MC group, DoMRE could significantly inhibit XOD activity in serum, liver, and small intestine of HUA model rats (*P* < 0.05, 0.01). It is suggested that DoMRE may reduce the production of UA by inhibiting XOD activity.

Compared with the NC group, the ADA activity in the liver of the MC group increased significantly (*P* < 0.01), and the ADA activity in the liver of both DoMRE dose groups was evidently reduced (*P* < 0.05) compared with the MC group. In addition, DoMRE-H significantly lowered small intestine ADA levels (*P* < 0.05) (Figures 3(d)–3(f)), whereas the serum ADA activity in all DoMRE dose groups showed a downward trend, but the difference was not statistically significant. It is suggested that DoMRE may reduce the production of UA by inhibiting ADA activity in the liver, thereby reducing UA.

### 3.4. DoMRE Promotes UA Excretion by Regulating Intestinal Transporters (ABCG2 and GLUT9) and Protein Levels in HUA Rats

**Figure 4:**
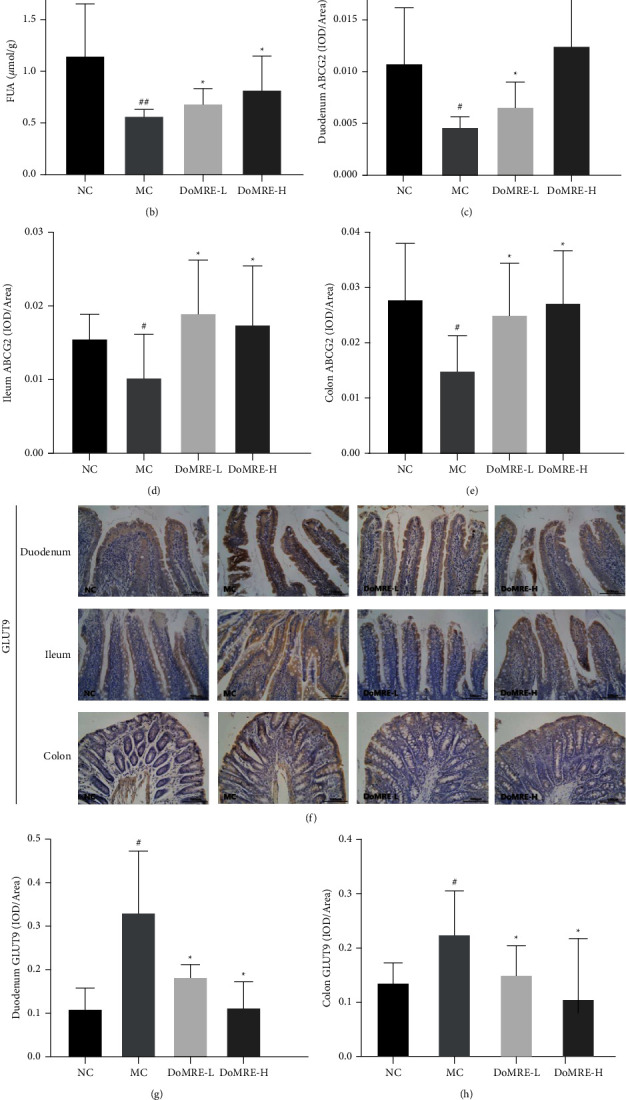
Effect of DoMRE on FUA levels and intestinal urate transporters ABCG2 and GLUT9 protein levels. (a) Intestinal ABCG2 IHC pictures (×400). (b) FUA levels. (c) Duodenum ABCG2 OD value. (d) Ileum ABCG2 OD value. (e) Colon ABCG2 OD value. (f) Intestinal GLUT9 IHC pictures (×400). (g) Duodenum GLUT9 OD value. (h) Colon GLUT9 OD value. (i) Ileum GLUT9 OD value. Semiquantitative analysis of ABCG2 and GLUT9 protein expressions in duodenum ileum, and colon were presented as IOD/Area. NC, normal control group; MC, model control group; DoMRE-L, low dose of DoMRE group; DoMRE-H, high dose of DoMRE group. The data were expressed as mean ± SD of 8 rats in each group. ^#^*P* < 0.05; ^##^*P* < 0.01, compared with NC group; ^^*∗*^^*P* < 0.05; ^^*∗∗*^^*P* < 0.01, compared with MC group.

Although the primary route for the excretion of UA is through the kidneys, extra-renal UA excretion through the enteral route becomes increasingly important in patients with decreased renal function [18, 19]. Therefore, we measured the FUA levels to evaluate the capacity of intestinal UA excretion in rats with HUA. As shown in Figure 4(b), after 12 weeks of modeling, the FUA levels in the MC group were significantly decreased compared to the NC group (*P* < 0.01); all doses of DoMRE treatment remarkably increased the FUA levels (*P* < 0.05) in the HUA rats, and it is suggested that continuous administration of DoMRE can promote the intestinal excretion of UA in HUA rats.

During UA excretion by the intestines, ABCG2 and GLUT9, which are expressed on the enterocytic apical and basement membranes of intestinal cells, play critical roles in urate excretion in the intestines [20, 21]. To elucidate the roles of these transporters and other relevant scaffold proteins in promoting intestinal UA excretion by DoMRE, we examined the changes in their concentrations in the intestine after DoMRE administration by IHC staining.

As shown in Figures 4(a)–4(i), compared with the NC group, the ABCG2 protein level in the duodenum, ileum, and colon of the MC group was significantly reduced (*P* < 0.05) and the GLUT9 protein levels (*P* < 0.05) were significantly elevated, while compared with the MC group, the ABCG2 level of rats in all DoMRE groups was significantly increased in duodenum, ileum, and colon tissues (*P* < 0.05); moreover, the two doses of DoMRE significantly decreased the GLUT9 protein expression in duodenum, ileum, and colon tissues (*P* < 0.05, 0.01), and it is suggested that DoMRE may regulate the expression of ABCG2 and GLUT9 proteins in the duodenum, ileum, and colon of HUA rats to promote UA excretion.

### 3.5. DoMRE Changes the Histopathological Changes in HUA Rats

**Figure 5:**
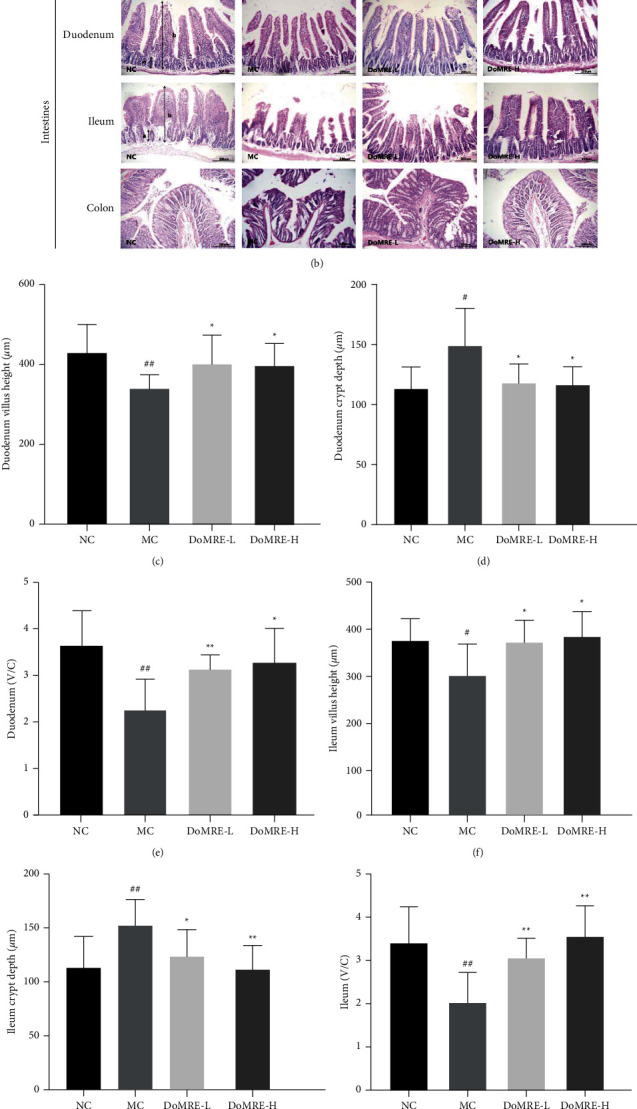
Effect of DoMRE on histopathological changes in the renal and intestine. (a) Renal tissues were stained with H&E at a magnification of 200x: (A) glomerulus significantly atrophy; (B) glomerular rupture; (C) the renal tubules swollen and deformed. (b) The duodenum, ileum, and colon were stained with H&E staining to observe crypt (A) and villus (B) at 200x. (c–h) The villus height (V), crypt depth (C), and V/C ratio in duodenum and ileum. IOD: integrated optical density; NC: normal control group; MC: model control group; DoMRE-L: low dose of DoMRE group; DoMRE-H: high dose of DoMRE group. The data were expressed as mean ± SD of 8 rats in each group. ^#^*P* < 0.05; ^##^*P* < 0.01, compared with NC group; ^^*∗*^^*P* < 0.05; ^^*∗∗*^^*P* < 0.01, compared with MC group.

After the renal tissue samples were stained with H&E, the results showed that the renal structure of the rats in the NC group was normal, as the renal cells were neatly arranged, and no inflammatory cell infiltration was observed in the renal interstitium. However, compared to the NC group, the renal tissue of the rats in the MC group showed various morphological changes, including significant atrophy in the glomerulus (a), glomerular rupture (b), and swelling and deformation of the renal tubules (c). Compared with the MC group rats, after 12 weeks of continuous administration, all doses of DoMRE could effectively ameliorate the glomerular atrophy and nephrons changes, indicating that the DoMRE could attenuate the renal pathology changes in the anthropomorphic unhealthy lifestyle-induced rat model of HUA (Figure 5(a)).

Intestinal histological changes are depicted in Figure 5(b); the epithelial cells of the duodenum, ileum, and colon showed clear margins, regular arrangement, and complete structure in the NC group. Compared with the NC group, the villus of duodenum and ileum became shorter, sparser, and thicker, and even some duodenum and ileum villus tissues were broken or shredded in model rats, and the colonic mucosa was severely damaged and crypts and glandular structures were severely damaged. As shown in Figures 5(c)–5(h), the villus heights of duodenum and ileum in the MC group were significantly decreased (*P* < 0.01), and the crypt depth of duodenum and ileum was remarkably increased (*P* < 0.01), whereas the villus height/crypt depth ratio of duodenum and ileum was significantly decreased in the anthropomorphic unhealthy lifestyle-induced rat model of HUA (*P* < 0.01).

Interestingly, after 12 weeks of continuous administration, all doses of DoMRE could significantly decrease the crypt depth of ileum and duodenum (*P* < 0.05, 0.01) and markedly elevate the villus height and the villus height/crypt depth ratio of the duodenum and ileum (*P* < 0.05, 0.01), whereas DoMRE could also improve the colonic mucosal injury and destruction of crypts and glands in the colonic tissues (Figures 5(c)–5(h)). It indicates that DoMRE could improve the intestinal lesions in HUA rats.

### 3.6. DoMRE Ameliorates Intestinal Histopathological Changes in HUA Model Rats by Protecting the Intestinal Barrier Function and Inhibiting the Secretion of Inflammatory Cytokines

**Figure 6:**
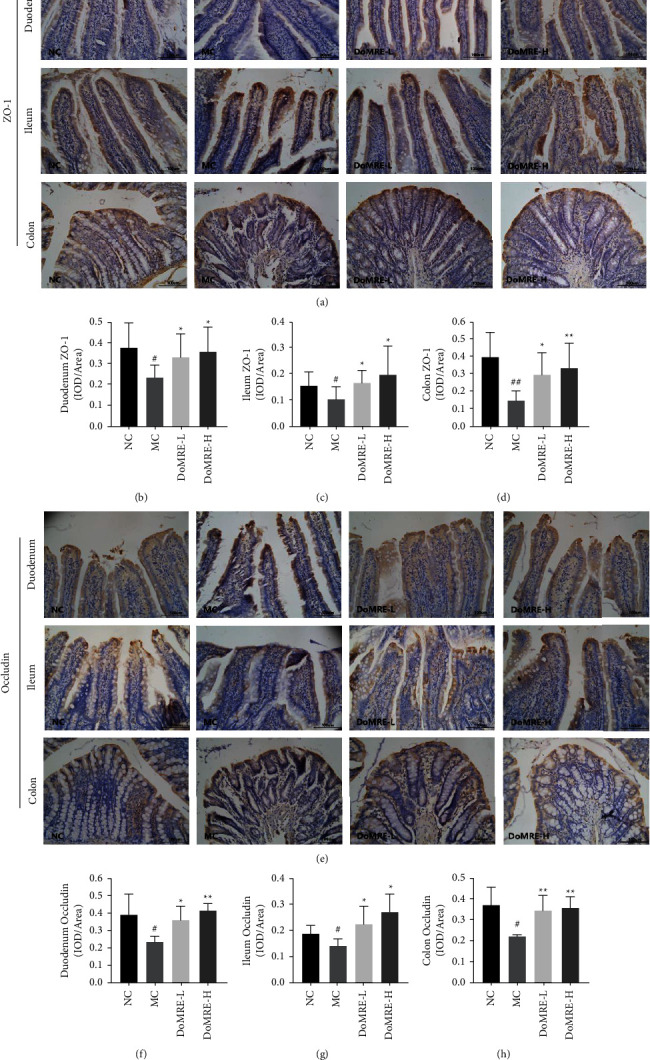
DoMRE restore the TJ proteins levels to protects the intestinal barrier function. (a) Intestinal ZO-1 IHC pictures (×400). (b) Duodenum ZO-1 OD value. (c) Ileum ZO-1 OD value. (d) Colon ZO-1 OD value. (e) Intestinal occludin IHC pictures (×400). (f) Duodenum occludin OD value. (g) Ileum occludin OD value. (h) Colon occludin OD value. Semiquantitative analysis of ZO-1 and occludin protein expressions in duodenum ileum, and colon were presented as IOD/area. NC, normal control group; MC, model control group; DoMRE-L, low dose of DoMRE group; DoMRE-H, high dose of DoMRE group. The data were expressed as mean ± SD of 8 rats in each group. ^#^*P* < 0.05; ^##^*P* < 0.01, compared with NC group; ^^*∗*^^*P* < 0.05; ^^*∗∗*^^*P* < 0.01, compared with MC group.

The intestinal epithelium has been demonstrated to be crucial for facilitating UA excretion in the intestine [22]. The intestinal epithelium and apical junctional complex constitute the intestinal barrier and function as the dynamic interface between the internal and external environments, which is crucial for maintaining intestinal homeostasis [23, 24]. As important indicators for evaluating the functional integrity of the intestinal mucosal barrier, ZO-1 and occludin were considered to be the two most important tight junction proteins within the intestinal mucosal barrier for enabling its function [25]. As exhibited in Figure 6, compared with the NC group, the expressions of ZO-1 and occludin proteins in duodenum, ileum, and colon of MC group were significantly reduced (*P* < 0.05, 0.01). Compared with the MC group rats, all doses of DoMRE treatment markedly elevated the ZO-1 and occludin proteins levels in the duodenum, ileum, and colon as compared to the MC group (*P* < 0.05, 0.01). These results indicated that DoMRE effectively restored the levels of tight junction proteins in the duodenum, ileum, and colon in the HUA rats, which enabled the barrier to repair and protect itself from the damage induced by the HUA.

**Figure 7:**
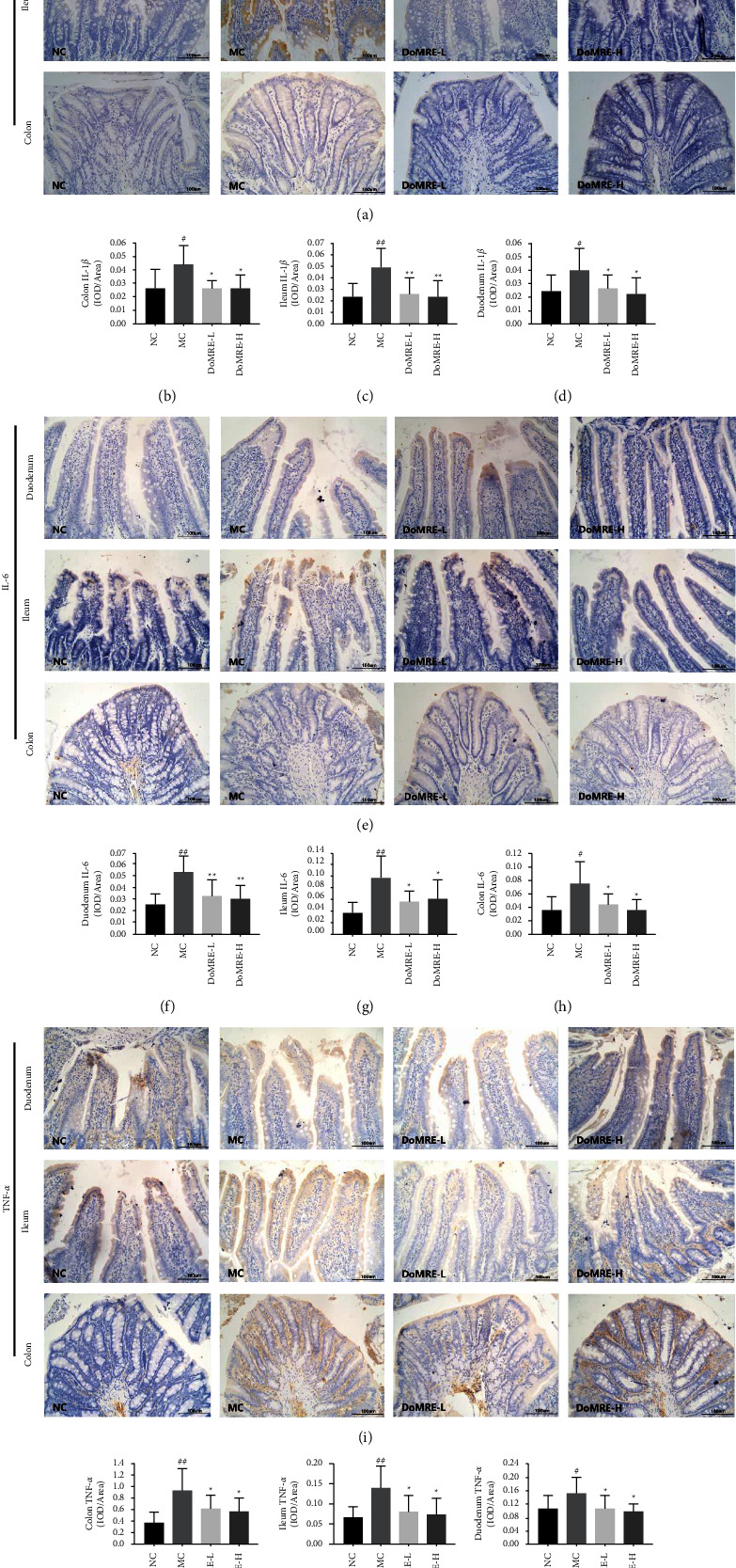
Effects of DoMRE on the inflammatory factors TNF-*α*, IL-6, and IL-1*β* of colonic mucosa in HUA rats. (a) Intestinal IL-1*β* IHC pictures (×400). (b) Colon IL-16 OD value. (c) Ileum IL-1*β* OD value. (d) Duodenum IL-16 OD value. (e) Intestinal IL-6 IHC pictures (×400). (f) Duodenum IL-6 OD value. (g) Ileum IL-6 OD value. (h) Colon IL-6 OD value. (i) Intestinal TNF-*α* IHC pictures (×400). (j) Colon TNF-5 OD value. (k) Ileum TNF-*α* OD value. (l) Duodenum TNF-5 OD value. Semiquantitative analysis of TNF-*α*, IL-6 and IL-1*β* protein expressions in duodenum ileum, and colon were presented as IOD/area. NC, normal control group; MC, model control group; DoMRE-L, low dose of DoMRE group; DoMRE-H, high dose of DoMRE group. The data were expressed as mean ± SD of 8 rats in each group. ^#^*P* < 0.05; ^##^*P* < 0.01, compared with NC group; ^^*∗*^^*P* < 0.05; ^^*∗∗*^^*P* < 0.01, compared with MC group.

Uric acid-induced inflammation can hinder intestinal mucosal barrier function [26]; thus, the inflammation status and changes in the intestinal structure are often regarded as obvious pathological characteristics of HUA [19]. We investigated the changes in the levels of the IL-1*β*, IL-6, and TNF-*α* inflammatory cytokines in the duodenum, ileum, and colon after DoMRE administration to the rats by IHC staining. As shown in Figure 7, compared with the NC group, the expression of IL-1*β*, IL-6, and TNF-*α* levels was significantly elevated in the duodenum, ileum, and colon of the MC group (*P* < 0.05, 0.01), whereas all doses of DoMRE treatment significantly reversed the elevations of IL-1*β*, IL-6, and TNF-*α* levels (*P* < 0.05, 0.01) in the duodenum, ileum, and colon of the anthropomorphic unhealthy lifestyle-induced HUA rats. The results indicated that DoMRE ameliorated intestinal histopathological changes in the HUA rat model by protecting the intestinal barrier function and inhibiting the secretion of inflammatory cytokines.

## 4. Discussion

SUA level is one of the hallmarks of HUA in animals. During the past decade, lifestyle and diet changes have caused an increase in prevalence of HUA in China; consequently, HUA has become a common metabolic disease, especially in China [27, 28]. Unhealthy lifestyle choices, such as irregular work and sleep schedules, consuming high-fat and high-sugar diets, excessive drinking, and mental stress are some of the main factors promoting the rising incidence of HUA. Consuming foods high in fat and protein in excess can lead to impairments in the body's purine biosynthesis, resulting in higher blood UA concentrations [29]. Excessive intake of fructose can accelerate the catabolism of adenosine, leading to the production of UA and causing HUA [29]. Alcohol consumption can increase lactic acid levels in the body, which inhibits the excretion of UA by the renal tubules, and promote purine decomposition, which directly increases SUA levels [30]. Chronic psychological stress can directly enhance the function of the hypothalamic-pituitary-adrenal axis (HPA axis) and sympathetic adrenal medulla, which affects purine metabolism and UA excretion, resulting in the continuous accumulation of UA in the body [9]. On the other hand, psychological stress increases UA production and induces oxidative stress by increasing xanthine oxidoreductase activity, resulting in oxidative damage caused by the accumulation of reactive oxygen free radicals in the body and affecting purine metabolism, as well as endothelial dysfunction, which affects UA excretion [31, 32].

In this study, we exposed rats to “chronic stress + drinking alcohol + feeding high sugar and high fat + potassium oxazine by oral gavage” for eight weeks to simulate unhealthy lifestyles of humans to establish a HUA rat model. As expected, exposure of the rat to the anthropomorphic unhealthy lifestyle for the eight-week period led to significantly increased SUA levels. After 12 weeks, the SCr levels in the MC group of rats were significantly higher than those in the NC group, validating this approach as a reproducible model of HUA in rats. These results demonstrated that DoMRE could effectively reduce UA levels, which is consistent with our previous study that showed that DoMRE reduced UA levels in HUA rats induced by a high-purine diet [12]. This model may be useful for future research to elucidate the causes and pathogenesis of metabolic disorders involving HUA and to evaluate new therapeutic candidates. Not only are the compositions of the primary chemical components of the stems and leaves of *Dendrobium officinale* similar but also these primary components provide a variety of pharmacological effects [33]. Combined with the results of this study, *Dendrobium officinale* leaves are worth studying further to identify possible therapeutic drugs for treating hyperuricemia.

In bodily fluids, UA mostly exists as urate, which is produced from hypoxanthine and xanthine oxidation by XOD during the metabolism of endogenous and exogenous purines and is excreted from the body via the urine and intestinal tract [1]. Therefore, the treatment of HUA mainly focuses on inhibiting UA production and promoting UA excretion [34]. XOD and ADA are vital enzymes that regulate the production of UA and are mainly expressed in the liver and intestine. ADA catalyzes the decomposition of adenosine into the hypoxanthine nucleoside inosine, which is then metabolized by nucleoside phosphorylase to generate hypoxanthine, the oxidation of which by XOD affords UA [35, 36]. Therefore, XOD and ADA are promising biological targets for reducing UA production. In this research, after administration of DoMRE to the rats, the XOD activity in the serum, liver, and intestine and the ADA activity in the liver and intestine were significantly reduced. These results suggested that chemical components of DoMRE inhibited the activity of XOD and ADA, thereby reducing UA levels in the rats by mitigating the synthesis of UA.

The excretion of UA from the body through urine serves as the main regulator of SUA levels and accounts for approximately 65% of all urate elimination [37], while intestinal excretion accounts for the remaining 35% [38]. However, in patients with reduced renal function, the gastrointestinal tract becomes the main route of uric acid excretion [18]. In the present study, after administration of DoMRE to the HUA rats, we observed a significant reduction in SUA levels, as well as increased UA excretion in the renal and intestine. In the rat model with reduced renal function, the excretion of UA through the intestine was particularly important.

Excretion of UA requires specialized transporters located on the surface of renal tubule cells and intestinal epithelial cells. Two of those transporters are ABCG2 and GLUT9, which participate in the transportation and reabsorption of UA in the kidneys and intestine [39, 40]. ABCG2 is expressed on the apical membrane in certain tissues, including the kidneys and intestine. Research has indicated that ABCG2 plays a crucial role in UA homeostasis by facilitating both renal and extra-renal UA excretion [41]. In particular, ABCG2 plays an important role in the excretion of UA from the intestine by facilitating up to one-third of the excretion of UA via this route [38]. GLUT9 is essential in urate reabsorption and is widely expressed in the kidneys and liver and expressed at lower levels in other tissues, including the small intestine and colon [42]. GLUT9 can transport UA into the renal tubules via renal tubular epithelial cells, which enables UA to enter circulation. Increasing evidence has shown that intestinal transporters, such as GLUT9, also play critical roles in intestinal urate excretion by mediating urate transport across enterocytes. For example, GLUT9 knockout mice displayed lower intestinal excretion of UA compared to normal mice [20]. In this study, we investigated the effects of DoMRE on the extra-renal elimination of urate by studying the changes in activities of relevant urate transporter proteins before and after administration of DoMRE to the HUA rats. The results indicated that, after treatment of the HUA rats with DoMRE, the expression of ABCG2 was significantly upregulated, while the expression of GLUT9 was significantly downregulated, in the intestines, which indicated that DoMRE regulated the expression of intestinal ABCG2 and GLUT9 proteins to promote intestinal UA excretion and inhibit the reabsorption of UA.

HUA can interfere with the epithelial integrity of the intestine, contributing to intestinal damage [26]; intestinal epithelial cell damage can further promote dysfunction of UA transport by the intestinal epithelial cells, which will lead to increase in SUA levels [43]. The villi of the small intestine are the structures on the surface of the intestinal epithelium that regulate absorption. The villi form structural units with intestinal crypts, which are tubular glands formed by the root epithelium of the villi at the lamina propria. The villi height, crypt depth, and their ratio (V/C) all can reflect the physiological function and injury degree of intestinal tissue [44]. The H&E staining patterns of the intestinal tissue harvested from the HUA rats showed that the duodenal and ileal villi in the HUA rat model were ruptured and sparsely arranged and contained partial shedding and lesions. Furthermore, the colonic mucosa was severely damaged, which allowed some inflammatory cells to infiltrate the mucosal lining, and the crypts and glandular structures were severely damaged. Semiquantitative results showed that the HUA rats had significantly lower duodenal and ileal villi heights (V), reduced V/C values, and higher ileal crypt depths (C) compared to the NC group. In addition, after the two different doses of the DoMRE, significant increases in the height of the duodenal and ileal villi were observed, as were reductions in the depth of the duodenal and ileal crypts and increases in the V/C values of the duodenum and ileum. These results indicated that DoMRE promoted the proliferation and differentiation of intestinal mucosal epithelial cells as well as restored the integrity of the intestinal mucosal barrier after damage from the HUA. Therefore, DoMRE appeared to be efficacious in maintaining the integrity of the intestinal barrier structure and function.

Tight junctions (TJs) between the intestinal epithelial cells are involved in the maintenance of the intestinal mucosal barrier function, and the abnormal intestinal UA excretion in HUA state is closely related to pathological changes of the intestinal barrier [24]. The intestinal mucosal barrier is the most important barrier in the intestine. TJs, mainly formed from transmembrane proteins such as occludin and ZO-1, provide a mechanical barrier that limits permeation through the epithelial lining [45]. In our study, the expression levels of ZO-1 and occludin in the duodenum, ileum, and colon of the HUA model rats were significantly reduced due to the HUA, indicating that the intestinal barrier was damaged. However, after administration of all doses of DoMRE, we observed significant increases in the expression levels of ZO-1 and occludin in the duodenum, ileum, and colon of the rats, suggesting that the DoMRE functioned to protect and maintain the structural and functional integrity of the intestinal barrier.

According to our results, HUA promoted significant damage to the intestinal mucosal barrier, which increased permeation through the intestinal lining manifest and promoted intestinal inflammation through the increased production of inflammatory cytokines (such as IL-1*β*, IL-6, and TNF-*α*) [24], which further negatively affect the function of the intestinal mucosal barrier and epithelium [46]. These experiments revealed that TNF-*α* reduced the number of TJs by downregulating expression of occludin and ZO-1 in the intestinal tissues [47]. According to our research, in the HUA rat model induced by the anthropomorphic unhealthy lifestyle for 12 weeks, the expression levels of IL-1*β*, IL-6, and TNF-*α* were significantly elevated in the intestinal tissue. However, the expression of these inflammatory factors was significantly reduced in both of the DoMRE treatment groups compared to the MC group. These results also indicated that DoMRE ameliorated the damage to the intestinal barrier caused by the pro-inflammatory factors and improved the capacity of intestinal epithelial transport of UA by regulating the expression of ABCG2 and GLUT9 caused by damage to the intestinal epithelium.

## 5. Conclusion

In conclusion, the HUA rat model was successfully established by exposing the rats to “chronic stress + drinking alcohol + feeding high-sugar and high-fat diets + potassium oxazine (oral gavage)” to induce physiological changes in the rats consistent with unhealthy lifestyles of humans, such as irregular work and sleep schedules, high mental stress, overeating, and excessive drinking. This model engendered changes in the metabolism and pathogenesis of UA in the rats that were more similar to humans compared to other HUA models; thus, this model could be used to study and evaluate the therapeutic effects of drugs for HUA.

**Figure 8:**
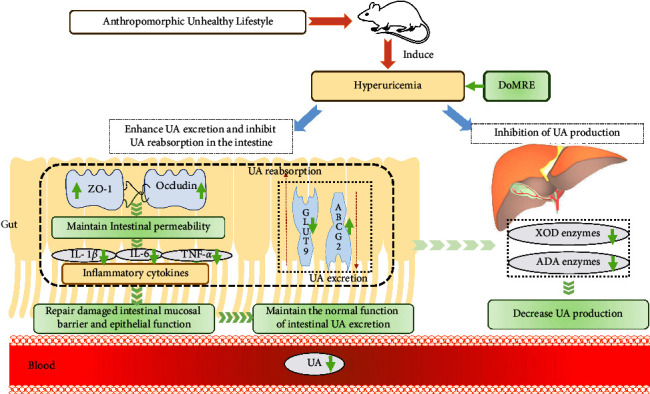
The mechanism of DoMRE against hyperuricemia. The yellow arrow represents hyperuricemia, whereas the green arrow represents DoMRE. On the one hand, DoMRE significantly upregulates the expressions of intestinal ZO-1 and occludin proteins, and reduce the intestinal barrier function damage caused by inflammatory factors, which cause the villus height significantly increased of intestinal tissue and a significant decrease in crypt depth of ileum, all of which lead to the improvement of the intestinal barrier function and the assurance of the normal function of intestinal UA excretion. Besides, the DoMRE treatment upregulate the expression of ABCG2 and downregulate the expression of GLUT9 to promote the intestinal UA excretion and purine intaking, ultimately reducing the SUA level. Besides, the DoMRE treatment upregulate the expressions of intestinal ZO-1 and claudin-1 proteins, to improve the intestinal barrier function, so as to ensure the normal function of intestinal UA excretion. Meanwhile, DoMRE can also inhibit XOD and ADA activity in intestine and liver to a certain extent, which ultimately lead to the reduction of the SUA level.

Meanwhile, DoMRE demonstrated anti-HUA effects by inhibiting the activity of XOD and ADA to reduce the production of UA and regulating the expression of intestinal urate transport-related transporters to enhance UA excretion and inhibit UA reabsorption in the intestine of the HUA rats. The mechanism of its possible anti-HUA effect is shown in Figure 8. DoMRE significantly upregulated the expression of intestinal ZO-1 and occludin proteins and mitigated damage to the intestinal barrier function caused by increased production of inflammatory factors due to HUA, which induced structural changes in the intestine, such as higher villi heights and decreased crypt depths in the ileum. Therefore, DoMRE was efficacious in restoring the intestinal barrier function and, therefore, intestinal UA excretion. Furthermore, DoMRE upregulated the expression of ABCG2 and downregulated the expression of GLUT9 to promote intestinal UA excretion. Lastly, DoMRE inhibited XOD and ADA activity in the intestine and liver to a certain extent, which ultimately led to a reduction in the SUA levels in the rats.

## Figures and Tables

**Table 1:** Chronic unpredictable stress protocol applied to SD rats for 12 weeks.

Day	Stressor (time)
1	Food deprivation (24 h)
2	Without stressor
3	Clip tail (30 min)
4	Food deprivation (24 h)
5	Overcrowding (24 h)
6	Clip tail (30 min)
7	Ice water swimming (5 min)
8	Without stressor
9	Overcrowding (24 h)
10	Wet bet (24 h)
11	Sleep deprivation (24 h)
12	Food deprivation (24 h)
13	Ice water swimming (5 min)
14	Ice water swimming (5 min)
15	Sleep deprivation (24 h)
16	Overcrowding (24 h)
17	Food deprivation (24 h)
18	Overcrowding (24 h)
19	Overcrowding (24 h)
20	Clip tail (30 min)
21	Sleep deprivation (24 h)
22	Overcrowding (24 h)
23	Without stressor
24	Overcrowding (24 h)
25	Without stressor
26	Overcrowding (24 h)
27	Sleep deprivation (24 h)
28	Clip tail (30 min)
29	Sleep deprivation (24 h)
30	Wet bet (24 h)
31	Wet bet (24 h)
32	Overcrowding (24 h)
33	Wet bet (24 h)
34	Ice water swimming (5 min)
35	Without stressor
36	Clip tail (30 min)
37	Wet bet (24 h)
38	Clip tail (30 min)
39	Sleep deprivation (24 h)
40	Food deprivation (24 h)
41	Wet bet (24 h)
42	Overcrowding (24 h)
43	Without stressor
44	Ice water swimming (5 min)
45	Overcrowding (24 h)
46	Wet bet (24 h)
47	Overcrowding (24 h)
48	Sleep deprivation (24 h)
49	Overcrowding (24 h)
50	Wet bet (24 h)
51	Without stressor
52	Sleep deprivation (24 h)
53	Ice water swimming (5 min)
54	Clip tail (30 min)
55	Sleep deprivation (24 h)
56	Clip tail (30 min)
57	Food deprivation (24 h)
58	Clip tail (30 min)
59	Sleep deprivation (24 h)
60	Food deprivation (24 h)
61	Without stressor
62	Wet bet (24 h)
63	Wet bet (24 h)
64	Clip tail (30 min)
65	Wet bet (24 h)
66	Food deprivation (24 h)
67	Ice water swimming (5 min)
68	Food deprivation (24 h)
69	Ice water swimming (5 min)
70	Overcrowding (24 h)
71	Without stressor
72	Food deprivation (24 h)
73	Ice water swimming (5 min)
74	Food deprivation (24 h)
75	Clip tail (30 min)
76	Without stressor
77	Clip tail (30 min)
78	Food deprivation (24 h)
79	Sleep deprivation (24 h)
80	Ice water swimming (5 min)
81	Wet bet (24 h)
82	Ice water swimming (5 min)
83	Ice water swimming (5 min)
84	Overcrowding (24 h)

**Table 2:** Alcohol consumption gradient scale.

Day	1∼4 (%)	5∼8 (%)	9∼12 (%)	13∼15 (%)	16∼20 (%)	21∼25 (%)	26∼30 (%)	After 30 days (%)
Alcohol volume fraction	4	8	12	16	19	21	22	22

## Data Availability

The datasets used and/or analyzed during the current study are available from Dr. Lin-Zi Li on reasonable request.

## Abbreviations

DoMRE: Macroporous resin extract of *Dendrobium officinale* leaves
UA: Uric acid
HUA: Hyperuricemia
SUA: Serum uric acid
UUA: Urine uric acid
FUA: Fecal uric acid
FEUA: Fraction excretion of uric acid
ADA: Adenosine deaminase
XOD: Xanthine oxidase
GLUT9: Glucose transporter 9
ABCG2: ATP-binding cassette superfamily G member 2
ZO-1: Zonula occludens 1
IL-1*β*: Interleukin-1*β*
TNF-*α*: Tumor necrosis factor-*α*.
